# 20 Years of Fatty Acid Analysis by Capillary Electrophoresis

**DOI:** 10.3390/molecules190914094

**Published:** 2014-09-09

**Authors:** Marcone Augusto Leal de Oliveira, Brenda Lee Simas Porto, Isaura Daniele Leite Faria, Patricia Lopes de Oliveira, Patricia Mendonça de Castro Barra, Renata de Jesus Coelho Castro, Renata Takabayashi Sato

**Affiliations:** Grupo de Química Analítica e Quimiometria (GQAQ), Departamento de Química, Instituto de Ciências Exatas, Universidade Federal de Juiz de Fora, Juiz de Fora, MG 36036-900, Brazil

**Keywords:** fatty acids, capillary electrophoresis, review

## Abstract

A review taking into account the literature reports covering 20 years of fatty acid analysis by capillary electrophoresis is presented. This paper describes the evolution of fatty acid analysis using different CE modes such as capillary zone electrophoresis, non-aqueous capillary electrophoresis, micellar electrokinetic capillary chromatography and microemulsion electrokinetic chromatography employing different detection systems, such as ultraviolet-visible, capacitively coupled contactless conductivity, laser-induced fluorescence and mass spectrometry. In summary, the present review signals that CE seems to be an interesting analytical separation technique that is very useful for screening analysis or quantification of the usual fatty acids present in different matrices, offering short analysis times and a simple sample preparation step as inherent advantages in comparison with the classical methodology, making it a separation technique that is very attractive for quality control in industry and government agencies.

## 1. Introduction

Fatty acids (FAs) comprise carboxylic acids that are aliphatic and typically linear, and monocarboxylic acids with long hydrocarbon chains which are represented by the general form RCOOH, where R represents the carbon chain. They can be classified according to carbon chain length as: short chain FAs (SCFAs): from 2 to 4 carbon atoms; medium chain FAs (MCFAs): from 6 to 10; long chain FAs (LCFAs): from 12 to 20 and very long chain FAs (VLCFAs): up to 22 carbon atoms. In addition, they can be further categorised according to their degree of saturation into saturated fatty acids (SFAs), which do not contain any double bonds or other functional groups along the chain; monounsaturated fatty acids (MUFAs), which contain one double bond in the chain, and polyunsaturated fatty acids (PUFAs), which contain more than one double bond in the chain [1,2]. They are widely found in Nature (e.g., in food products, vegetable oils, and living organisms, *etc.*), with chain lengths containing an even number of carbon atoms and are the basic components that are most naturally present in the lipids of animals and plants. The broadest definition includes all chain lengths, but most natural FAs have chain lengths ranging between C4 and C22 [1,3].

The physical and chemical characteristics of FAs are associated with the carbon chain length, number and position of double bonds and *cis-trans* isomer configurations. Thus, FA are soluble in organic solvents, weakly soluble in water in their non-dissociated form and moderately hydrophilic as salts [4]. A *cis*-double bond in a FA introduces a 30° bend in the alkyl chain, tending to result in looser packing in membranes or crystal structures. *cis*-Monoenoic VLCFAs have a relatively high melting point, but the more common C18 monoenes tend to be liquids at room temperature. Triacylglycerols (or oils and fats) containing high proportions of monoenoic FAs are usually liquid at ambient temperature [5]. On the other hand, *trans*-fatty acids (TFAs) can be defined as monounsaturated or polyunsaturated FAs with carbon-carbon double bonds in the *trans* configuration or non-conjugated ones which normally have higher melting temperatures when compared with the *cis* configuration [6]. *Trans* isomers may be produced during industrial processing of unsaturated vegetable oils (catalytic hydrogenation) or by ruminant animals in their gastrointestinal tract (biohydrogenation).

**Figure 1:**
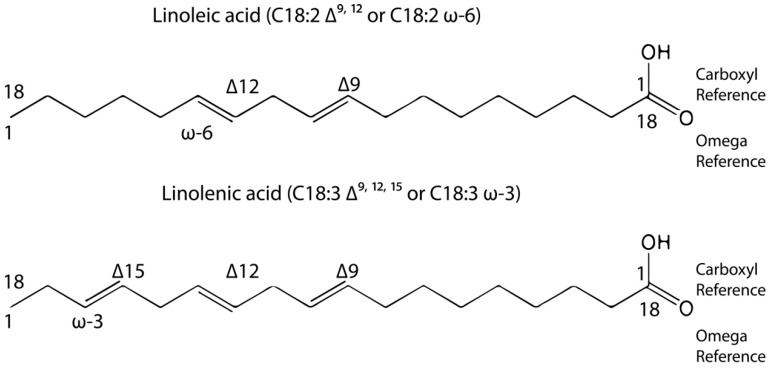
IUPAC and omega nomenclatures.

According to the International Union of Pure and Applied Chemistry (IUPAC) [7], the carboxyl carbon is denoted by the number one, and other positions in the chain are denoted with reference to this. This nomenclature rule indicates the number of carbons and the positions of the double bonds, counting from the carboxyl carbon that is considered the reference (Δ) [7]. However, in order for studies involving physiological research to be practical [7], the omega (ω) nomenclature was introduced. Thus, omega nomenclature takes into account the number of carbons and the position of the double bond nearest to the last carbon atom of the chain, taking the carbon atom from the carboxylic group as the reference. Figure 1 shows practical examples of the use of omega nomenclature. In the present case, linoleic and linolenic acids were classified as omega 6 and 3, respectively, because the first double bond is located at carbons number six and three when considering the numbering of the carbon chain from left to right.

FAs can further be incorporated into more complex molecules, used for energy production through β-oxidation in the mitochondria or stored as an energy reservoir in the form of triglycerides (TG) in lipid droplets [8]. The SCFAs are produced by fermentation of carbohydrates and proteins ingested from the diet, such as fibres, prebiotics and probiotics. This fermentation is performed in the large intestine by anaerobic bacteria, especially of the genera *Bifidobacterium* and *Lactobacillus*. The bacterial growth is beneficial to itestinal health and, at the same time, inhibits the growth of pathogenic bacteria. The increased concentration of SCFAs may also produce benefits for constipation symptoms. That is because these FAs are able to increase stool bulk and reduce intestinal transit time [9].

Diets containing MCFAs are of nutritional interest because they are more easily absorbed by the intestine than those rich in LCFAs. MCFAs are absorbed directly into the bloodstream and carried to the liver, and glucose does not significantly incorporate the lipoproteins to be transported. Tholstrup *et al.* performed a comparative study of diets rich in MCFAs and LCFAs on lipid profile and plasma glucose. The results showed that there is an increase of plasma total cholesterol, low density lipoprotein (LDL), very low density lipoprotein (VLDL), the ratio of LDL and HDL (high density lipoprotein), and total plasma triglycerides, as well as elevation of plasma glucose when a diet rich in MCFAs is considered [10].

Omega-6 LCFAs are biosynthesised from linoleic acid (18:2 omega-6) using a similar route and the same enzyme system as omega-3 FAs. LCFAs are also important for the maintenance of cellular membranes, because they are present in phospholipids and are also precursors in the synthesis of eicosanoids [11]. Due to the interrelationships of polyunsaturated FAs in the synthesis of eicosanoids and the existence of a high content of docosahexaenoic acid (DHA-C22: 6 omega-3) in the brain and retina, it is suggested that this acid plays an important role in proper functioning of the nervous and visual systems [12]. After ingestion, the FAs, once absorbed by cells and tissues, can be desaturated and elongated to other long-chain PUFAs. The processes of elongation and desaturation of linoleic and α-linolenic acids occur in animals and slowly in men, resulting in various metabolites such as DHA and eicosapentaenoic acid (EPA—20:5 omega 3). Thus, it is postulated that the preferential accumulation of DHA in this tissue is probably a consequence of a process of up-take, synthesis and esterification of FAs that is very effective in neuronal cells [13]. Finally, saturated or unsaturated VLCFAs are essential lipids whose functional variety is qualified by variation in their chain length and degree of unsaturation. DHA can perform beneficial functions by inhibiting the formation of aggregates of β-amyloid peptide in Alzheimer's disease, which are as deleterious as in Parkinson disease and Amyotrophic Lateral Sclerosis, making these FAs induce the formation of protein aggregates [14].

The classical FA analysis method is gas chromatography coupled with a flame ionisation detector (GC-FID) [15], which is a good choice for food labelling due to its accuracy, convenience, and sensibility. The GC-FID method requires preliminary lipid fraction extraction from the sample followed by derivatisation reaction into fatty acid methyl esters (FAMEs). Thus, the use of anhydrous methanol in the presence of an acidic catalyst or *N*,*N*-dimethylformamide dimethyl acetal in the presence of pyridine is very usual, although other possibilities can be contemplated [15,16,17,18,19,20]. Many methods of lipid fraction extraction may be used. However, the most common extraction methods are: Folch (chloroform/methanol in proportion of 2:1) [17], Bligh and Dyer (chloroform/methanol/water in proportions of 1:2:0.8) [18], Sheppard (ethanol/diethyl ether in proportions of 3:1) [19] and Hara and Radin (hexane/isopropanol in proportions of 3:2) [20].

Alternative methodologies such as Fourier-Transform Infrared (FTIR) [21], attenuated total reflection infrared spectroscopy (ATR-IR) [22,23], silver ion high performance liquid chromatography (Ag^+^-HPLC) [24,25], gas chromatography coupled with mass detector (GC-MS) [26], and capillary electrophoresis (CE) [27,28,29,30,31] have also been used. These methodologies sometimes offer short analysis time (CE, ATR-IR and FTIR), a non-destructive approach (ATR-IR and FTIR) and simplicity in the sample preparation step (CE, ATR-IR and FTIR), all of which are an advantage in comparison with the classical methodology.

Due to the importance of FAs in different biochemistry and chemistry processes, the current review has focused on demonstrating that FA analysis by CE can be a very interesting analytical approach. Thus, a brief discussion about the CE modes contemplated, as well as the background electrolyte (BGE) optimisation, operational conditions and the possibilities and features of the detection system used, will be described. Besides, as CE methodologies for FA analysis have inherent advantages, such as short analysis time and the absence of derivatisation reactions in the sample preparation step, in the present review will present the key reasons why the analysis of FAs by CE has attracted the interest of scientific community for applications in different areas during the last 20 years.

## 2. Separations in Different CE and Detection Modes

Traditionally, FA analysis by CE takes into account BGE features such as the use of organic solvent in order to avoid micelle formation among the FAs, the fact that the pH must be higher than 7.0 to promote carboxyl group dissociation (FAs have pKa values of about 5.0) and the need to make it possible to analyse FAs in anionic form under counter-electroosmotic flow (EOF) and cathodic EOF, the use of chromophoric species to promote the indirect detection of saturated FAs since they present low molar absorptivity in the UV range and presence of cyclodextrins [32], neutral surfactants or alcohols to improve the selectivity among the *cis* homologues or *cis-trans* isomers. In general, sample preparation is performed through basic saponification of the triacylglycerides in order to obtain dissociated FAs in the solution to introduce inside the capillary. Within this context, several methodologies, using different modes of CE under different detection possibilities containing intrinsic particularities, have been described in the literature. They will be discussed in the subsequent sections.

### 2.1. CE with Direct UV Detection

FA analysis by CE using direct UV detection (UV-DD) is possible if the analytes present one or more unsaturated FA in a linear carbon chain. In other words, the presence of a double bond results in a FA molar absorptivity that is sufficient to achieve a signal peak that is adequate for detection within the interval from 200 to 250 nm [33]. Moreover, the sensitivity of this detection mode, in general, is higher in comparison with indirect UV detection, which is the most frequently used approach.

Miksik and Deyl [34] used microemulsion electrokinetic chromatography (MEEKC) for the separation of saturated FAs derivatised as phenacyl esters and compared this to micellar electrokinetic chromatography (MEKC). The separations occurred in a 57 cm fused-silica capillary, at +15 kV, 30 °C and with UV detection under 243 nm, within an analysis time of 25 min. The microemulsions were prepared by mixing heptane (0.66% w/w), cholate (4.87% w/w) (selected as surfactant), *n*-butanol (6.55% w/w), and 10 mmol·L^−1^ borate buffer (87.93% w/w). The BGE used for MEKC separation was similar to that used for MEEKC; however, there was no addition of organic solvents. The separation was achieved for both modes, although the MEEKC approach was better than MEKC.

Bohlin *et al.* performed the separation of conjugated trienoic fatty acids (CTFAs) in seed oil using MEKC direct UV detection at 268 nm. The operational conditions were: fused silica capillary with an effective length of 50 cm (58 cm total length) and an internal diameter (i.d.) of 50 µm, voltage of +30 kV, cartridge temperature of 15°C and sample injection pressure of 50 mbar for 10s. The separation of seven isomers in about 15 min was achieved. The electrolyte system consisted of 24 mmol·L^−1^ sodium dodecyl sulphate (SDS), 20% acetonitrile (ACN), 40 mmol·L^−1^ borate (pH 9.2), 4 mmol·L^−1^ urea, 5 mmol·L^−1^ (*R*)-N-dodecoxycarbonylvaline [(R)-N-DOCV], 1 mmol·L^−1^ heptakis-(6-sulpho)-β-cyclodextrin (HS-β-CD) and 10 mmol·L^−1^ β-cyclodextrin (β-CD) [35].

In 2003, Tavares *et al.* [36] published a review describing applications in diverse matrices involving different CE modes. For FA analysis, a method using direct UV detection without the addition of cyclodextrins was described. In the present case, samples of enriched milk and fish oil were analysed qualitatively. In 2011, Porto *et al.* [29] adapted the method described to Tavares and co-workers for application in the separation of omega 3 FAs such as oleic acid (C18:1 *cis*-9), linoleic acid (C18:2 *cis 9,12*), α-linolenic acid (C18:3 *cis 9, 12, 15 alpha*), eicosapentaenoic acid (C20:5 *cis 5, 8, 11, 14, 17*), and docosahexaenoic acid (C22:6 *cis 4, 7, 10, 13, 16, 19*) in enriched chicken eggs using CE under direct UV detection at 200 nm, within an analysis time of 10 min. The operational conditions were: fused silica capillary of 40 cm effective length (48.5 cm total length) of 50 µm i.d., +27 kV, cartridge temperature of 27 °C and sample introduction at a pressure of 25 mbar for 5 s. The BGE used consisted of: 12.0 mmol·L^−1^ sodium tetraborate (pH ~ 9.2), 12.0 mmol·L^−1^ of polyoxyenthylene 23 lauryl ether (Brij 35), 17% acetonitrile (ACN), and 33% methanol (MeOH).

Liu and co-workers carried out the separation of seven isomers of Conjugated Linoleic Acid (CLA) using MEKC under direct UV detection at 231 nm. The operational conditions were: fused silica capillary with an effective length of 50 cm (61 cm total length) with a 100 µm i.d., +30 kV, cartridge temperature of 15°C and sample introduction at a pressure of 0.5 psi for 5 s. The isomer separation (*cis-9*, *cis-11*-CLA, *cis-9*, *trans*-*11*-CLA, *trans-9*, *trans-11*-CLA, *trans-10*, *cis-12*-CLA, *cis-11*, *cis-13*-CLA, *cis-11*, *trans-13*-CLA and *trans-11*, *trans-13*-CLA) was achieved in about 15 min. The BGE consisted of 4% (w/v) β-CD, 54 mmol·L^−1^ SDS, 80 mmol·L^−1^ borate (pH ~ 9.0), 8 mol L^−1^ urea and 4% (v/v) ethanol [37]. In a later work, Liu *et al.* also used the same methodology to perform screening study of bacterial strains able to synthesize CLA [38].

Soliman *et al.* developed an MEKC methodology for the simultaneous separation of fifteen omega 3s and six FAs in food samples. The operational conditions were: fused silica capillary with a total length of 60 cm, with a 50 µm i.d., +25 kV and sample introduction at a pressure of 0.5 psi for 5 s, under direct UV detection at 214 nm. The omega separation was as follows: α-linolenic acid (C18:3 n-3), docosatrienoic acid (C22:3 n-3), eicosapentaenoic acid (C20:5 n-3), docosapentaenoic acid (C22:5 n-3), docosahexaenoic acid (C22:6 n-3), linoleic acid (C18:2 n-6), α-linolenic acid (C18:3 n-6), nonadecadienoic acid (C19:2 n-6), 11,14-eicosadienoic acid (C20:2 n-6), dihomogamma-α-linolenic acid (C20:3 n-6), arachidonic acid (C20:4 n-6), docosadienoic acid (C22:2 n-6), docosatetraenoic acid (C22:4 n-6), docosapentaenoic acid (C22:5 n-6) and 11,14,17-eicosatrienoic acid (C20:3 n-3); this was performed within an analysis time of 50 min. The BGE consisted of 50 mmol·L^−1^ of SDS, 10% ACN , 40 mmol·L^−1^ of borate (pH ~ 9.5), 6 mmol·L^−1^ of urea, 5 mmol·L^−1^ (*R*)-N-dodecoxycarbonylvaline) [(R)-N-COAD] and 10 mmol·L^−1^ β-CD [39].

Table 1 shows a summary of the work described in the literature considered FA analysis by CE using direct UV detection.

### 2.2. CE with Indirect UV Detection

Saturated FAs present low molar absorptivity in the UV-Vis band, and thus they are analysed by CZE under indirect UV detection (UV-ID), which is based on the addition of a chromophoric agent or buffer chromophore into the BGE. Ideally, the selected chromophore must have high molar absorptivity at the working wavelength, effective mobility close to the analytes in order to reduce electromigration dispersion and a negative charge [40]. Among the chromophores used for CZE FA analysis under UV-ID, sodium dodecylbenzenesulphonate (SDBS) [28,30,31,41,42,43,44,45] and adenosine monophosphate (AMP) [27,46,47] have been most frequently used. Thus, in general, for the saturated FA analyses, a baseline presents high molar absorptivity, and when the separated FA crosses in front of the detection window, the molar absorptivity decreases and generates a negative peak.

Gutnikov *et al.* [48] developed a CZE methodology with normal and reversed EOF by indirect UV detection. They used 30% acetone, a cationic surfactant, and 3.5-dinitrobenzoic acid as BGE. The influence of the concentration of various organic solvents on the magnitude and direction of the EOF and on the solubility of the FA solutes were also investigated. This method was applied for the analysis of C_12_ at C_18_ acids in fat hydrolysates from butter and palm oil, and the separation was achieved within an analysis time of 12 min.

Erim *et al.* [41] analysed FAs in butter samples through ethanolic alkaline saponification and posterior dissolution of the vaporised residue in the BGE. The study resulted in baseline FA separation using BGE consisting of 10 mmol·L^−1^ of SDBS, 50% of ACN and 30 mmol·L^−1^ of Brij 35, under indirect UV detection at 198 nm. It is important to stress that the separation obtained by Erim *et al.* was more efficient than that obtained by Gutnikov; however, the analysis time for the method developed by Erim was higher, taking around 20 min to complete separation. One of the factors that influences the time of analysis is the type of surfactant used. Roldan-Assad [40] published the first papers dealing with the CZE separation of carboxylic acid homologues, and suggested conditions that were simply adapted from those derived from the UV transparent small inorganic or organic anions. These conditions generally involved the use of a cationic surfactant to reverse the electroosmotic flow and hence shortened the analysis time.

**Table 1:** Experimental condition, BGE and FA in different samples using UV-DD with different CE modes. CZE: Capillary zone electrophoresis.

Samples	FA	BGE and Experimental Condition	CE Mode and Detection	Year	Ref.
Mix standard	Saturated C_2_ at C_20_	heptane (0.66% w/w), cholate (4.87% w/w), n-BuOH (6.55% w/w) and 10 mmol·L^−1^ borate buffer (87.93% w/w); 30 °C, 15 kV	MEEKC-UV-DD (λ = 243 nm)	1998	[34]
Seed oils	CTFAs	24 mmol·L^−1^ SDS, 20% ACN, 40 mmol·L^−1^ borate (pH 9.2), 4 mmol·L^−1^ urea, 5 mmol·L^−1^ de [(R)-N-DOCV], 1 mmol·L^−1^ HS-β-CD and 10 mmol·L^−1^ β-CD; 15 °C, 30 kV	CZE-UV-DD (λ = 268 nm)	2003	[35]
Fish oil and enriched milk	C_16:0_, C_18:1c_, C_18:2cc_, C_18:3ccc_, C_20:5ccccc_ and C_22:6cccccc_	10 mmol·L^−1^ tetraborate (pH 9.2), 10 mmol·L^−1^ of Brij 35, 25% ACN and 25% MeOH; 25 °C, 30 kV for DD	CZE-UV-DD (λ = 200 nm for DD)	2003	[36]
Mix standard	9c, 11c-CLA, 9c, 11t-CLA, 9t, 11t-CLA, 10t, 12c-CLA, 11c, 13c-CLA, 11c, 13t-CLA and 11t, 13t-CLA	4% (w/v) β-CD, 54 mmol·L^−1^ SDS, 80 mmol·L^−1^ borate (pH 9.0), 8 mol L^−1^ urea and 4% (v/v ) EtOH; 15 °C, 30 kV	CE-DAD-DD (λ = 231 nm)	2005	[37]
Enriched chicken eggs	C_18:1 c_, C_18:2 cc_, C_18:3ccc_, C_20:5 ccccc_ and C_22:6 cccccc_	12 mmol·L^−1^ tetraborate (pH 9.2), 12 mmol·L^−1^ of Brij 35, 17% ACN and 33% MeOH; 27 °C, 27 kV	CZE-UV-DD (λ = 200 nm)	2011	[29]
Bacterial strains	9c, 11c-CLA, 9c, 11t-CLA, 9t, 11t-CLA, 10t, 12c-CLA, 11c, 13c-CLA, 11c, 13t-CLA and 11t, 13t-CLA	4% (w/v) β-CD, 54 mmol·L^−1^ SDS, 80 mmol·L^−1^ borate (pH 9.0), 8 mol L^−1^ urea and 4% (v/v) EtOH; 15 °C, 30 kV	CE-UV-DD (λ = 231 nm)	2012	[38]
Oil and meat	C_18:3 n-3_, C_22:3 n-3_, C_20:5 n-3_, C_22:5 n-3_, C_22:6 n-3_, C_18:2 n-6_, C_18:3 n-6_, C_19:2 n-6_, C_20:2 n-6_, C_20:3 n-6_, C_20:4 n-6_, C_22:2 n-6_, C_22:4 n-6_, C_22:5 n-6_ and C_20:3 n-3_	50 mmol·L^−1^ SDS, 10% ACN, 40 mmol·L^−1^ borate (pH 9.5), 6 mmol·L^−1^ urea, 5 mmol·L^−1^ [(R)-N-COAD] and 10 mmol·L^−1^ β-CD; 25 kV	CZE-UV-DD (λ = 214 nm)	2013	[39]

It is important to stress that one of the concerns of authors when CE began to be applied to FA analysis was the need to understand the separation mechanisms [46,49]. Thus, the variation in EOF and other parameters such as different sizes of capillaries, types of solvents and reagents and their implications regarding the solubility of analytes in the BGE and viscosity were investigated. Within this context, Collet and Gareil [50] presented work using MEKC under indirect UV detection at 254 nm to simultaneously analyse saturated and unsaturated FAs (from C14:0 to C20:0) with an analysis time of less than 5 min. Thus, the BGE consisted of 10 mmol·L^−1^ of Tris and 5 mmol·L^−1^ of *p*-methoxybenzoate (chromophore) (pH ~ 8.1), 10 mmol·L^−1^ of Brij 35 and 50% of methanol (MeOH).

Roldan-Assad *et al.* [40] performed CZE-UV FA analysis in coconut oil using BGE consisting of 20 mmol·L^−1^ of Tris-*p*-anisate chromophoric buffer (pH ~ 8.2), 1.0 mmol·L^−1^ of trimethyl-β-cyclodextrin, (TM-β-CD), and 40% of MeOH, under indirect detection at 270 nm. In this paper, the authors discussed, in detail, the improvement of FA solubility and efficiency when cyclodextrin concentrations increased due to FA inclusion inside a cyclodextrin cavity.

Oliveira *et al.* [31] optimised a CZE-UV methodology for the simultaneous analysis of majority saturated and unsaturated FAs in soybean oil, olive oil and margarine enriched using 5.0 mmol·L^−1^ phosphate electrolyte buffer (pH ~ 7.0), 4.0 mmol·L^−1^ of SDBS, 4.0 mmol·L^−1^ of DM-β-CD and 45% of ACN under indirect UV detection at 224 nm, within an analysis time of 8 min. Using the same BGE, Tavares *et al.* [36] also analysed FA in oils and fats in parenteral feed formulation.

In 2003, Tadaumi *et al.* [47] described a CZE-UV method for the simultaneous analysis of saturated and unsaturated FAs in olive oil, rice bran oil and flaxseed oil using a BGE consisting of 40 mmol·L^−1^ Tris buffer and 2.5 mmol·L^−1^ of AMP in MFN-dioxane-water (5:4:1 v:v:v), with an analysis time of 24 min. The authors also performed analyses of LCFAs and VLCFAs in salmon samples with the same BGE and experimental conditions, simply varying the ratio of solvents used; that is, AMP in MFN-dioxane-water (4:6:1 v:v:v). Despite the long analysis time of 50 min, the simultaneous separation of seven saturated and unsaturated FA was achieved. 

Another study considering free FA analysis in olive oil by CZE under indirect UV detection was performed by Balesteros *et al.* [43]. In the present case, the acidity expressed in oleic acid was determined by using BGE consisting of 15 mmol·L^−1^ of phosphate buffer (pH ~ 7.0), 4 mmol·L^−1^ of SDBS, 10 mmol·L^−1^ of Brij 35, 2% of 1-octanol and 45% of ACN. In this work, the sample preparation step was performed through hot ethanolic extraction. Also, the authors performed a study in order to solve the problems related to irreversible adsorption on the inner wall of the capillary, which resulted in band distortion and low reproducibility. Another problem pointed out by the authors was the low resistance of the polyamide to organic solvents. In order to investigate this further, the author left a 10 cm coated capillary polyamide into the BGE solution for one week and observed the polyamide external coating degradation by microscope. This result confirmed that the coating was partially dissolved in the BGE, and the polymer gradually adhered to the inner wall of the capillary, significantly disrupting the separation profile. A stopgap measure proposed and used by Balesteros *et al.* was the removal of 1.5 cm of the outer polyamide coating from the ends in order to prevent the electrolyte contact with polyamide. However, this procedure is not attractive because it makes the capillary more susceptible to breaking. Thus, the boundary condition was to use a fused silica capillary containing external coating consisted of fluoro-polymer (TSH), which is more resistant to abrasion and capillary solvents according to that which was described by Barra *et al.* [44].

Vergara-Barberan and co-workers [51] analysed saturated and *cis*-unsaturated FAs in several samples of vegetable oil (avocado, corn, extra virgin olive, hazelnut and soybean) with the objective of classifying them and also checking for possible fraud through a chemical-statistical model. The BGE consisted of 10 mmol·L^−1^ of Tris/*p*-hydroxybenzoate (PHB) buffer (pH ~ 8.8), 80 mmol·L^−1^ of Brij 98, 40% of ACN, 10% of 2-propanol and a wavelength of 254 nm. The authors tested different Brij and concluded that despite generating an increase in the migration time of FA, Brij 98 ensured an improvement in peak resolution; therefore, this was selected.

Drange *et al.* and [52] reported the work which attempted to analyse saturated and unsaturated FA in a sample oil from fish in nature, but obtained only one broad peak in the electropherogram. Thus, the fish oil sample was submitted to the hydrogenation reaction and then analysed again, now containing only saturated FAs, using a BGE composed of 2.5 mmol·L^−1^ of antraquinone-2-carboxylic acid (AQCA) and MFN in 40 mmol·L^−1^ of Tris-dioxane (3:1, v*:*v) and a wavelength of 264 nm. The analysis lasted for about 16 min and separated five saturated FAs.

Another work which applied a similar system to that used by Tadaumi was the study performed by Banore *et al.* [27]. This was the first article that analysed FAs in samples of peanut seeds by CE. In this study, the authors used a BGE consisting of 40 mmol·L^−1^ of Tris, 2.5 mmol·L^−1^ of AMP and 7.0 mmol·L^−1^ of α-CD in MFN-dioxane-water at proportion of 5:3:2 v:v:v and wavelength of 254 nm. The contribution of Banore and collaborators was the addition of α-CD, which functioned as a modulator for selectivity, increasing the effective FA mobility and reducing the analysis time by half.

An interesting methodology for analysing FAs was developed in 2003 when Oliveira *et al.* [53] who introduced the use of 1-octanol in BGE to achieve the separation of *cis-trans* isomers by CZE-UV. In this paper Brazil nut oil was submitted to catalytic hydrogenation in order to monitor the *trans*-FA formation. The oils in nature and those derived from the hydrogenation reaction were analysed by CZE using an electrolyte consisting of 15 mmol·L^−1^ of phosphate buffer (pH ~ 7.0), 4.0 mmol·L^−1^ of SDBS, 10 mmol·L^−1^ of Brij 35, 2% of 1-octanol and 45% of CAN with a wavelength of 224 nm. In this work, the focus of hydrogenation was a concern for the separation of *cis-trans* isomers present in processed foods. Later, this same BGE, after some optimisation was successfully applied to the analysis of food, fodder and biological samples [28,30,42,44,54,55].

In 2011, Lima *et al.* [45] reported FA analysis in cosmetic formulations containing Brazil nut oil using the electrolyte containing 12.5 mmol·L^−1^ of sodium tetraborate (STB), 12.5 mmol·L^−1^ of Brij 35 and 12.5 mmol·L^−1^ of SDBS, 35% of ACN, a wavelength of 254 nm and an analysis time of about 9 min.

Most of the papers presented in this section used CZE with indirect UV detection for the analysis of FA in food samples. However, there are some papers that have not examined actual samples and focused more on technical aspects, such as the influence of solvents and capillary size, and other points that may interfere with FA separation. Table 2 presents the scenery of FA methodologies using different modes of CE under indirect UV detection.

### 2.3. CE with Laser-Induced Fluorescence Detection

Laser-induced fluorescence detection (LIF) has been shown to be one of the most sensitive methods available for detection in CE (CE-LIF) [56]. As FAs generally lack suitable chromophores or fluorophores, detection by LIF requires derivatisation [57].

Gallaher *et al.* [56], presented in 1999 a synthesis and characterisation of polymethine cyanine near-infrared label for the derivatisation and determination of FAs separated by CE-LIF. This fluorophore was designed for coupling with the carboxylic group of the FAs. The separation was achieved with a bare fused-silica capillary (50 μm internal diameter), and total and effective lengths of 79 cm and 45 cm, respectively. The other analysis conditions were normal polarity, voltage +25 kV, electrokinetic injection (3 s, 10 kV), LIF detection excitation at 780 nm and ambient temperature. The electrolyte was methanol with 12.5 mmol·L^−1^ tetraethylammonium chloride. The FAs identified, with a mixture of standards, were: C3:0, C5:0, C6:0, C10:0, C12:0, C18:1 and C16:0. All peaks had good resolutions, except for the peaks of C12:0 and C18:1, which migrated together. Peak identities were confirmed by the injection of individual standards and by spiking the mixture with aliquots of individual standards. The total analysis time was approximately 11 min.

LCFAs and mycobacterial FAs were determined by Brando *et al.* [57] using CE-LIF. Analysis conditions were bare fused-silica capillary (50 μm internal diameter) and an effective length of 47 cm, normal polarity, +30 kV, hydrodynamic injection of 0.5 psi pressure for 5 s, LIF detection excitation at 488 nm and emission filter of 520 nm, with a temperature of 25 °C. The running electrolyte consisted of 25 mmol·L^−1^ aqueous sodium borate and 30% of ACN at pH 9. The fluorophore reagent employed was 4-aminofluorescein (AF) which interacts with the carboxyl groups of the FAs. Under these conditions, it was possible to separate and identify C19:0, C18:0, C16:0 with C14:0 (internal standard) derivatised at 6 min approximately, and in another run, these were separated and identified as C19:0, C18:1c and C16:0 in less than 6 min. The confirmation of peaks was performed by standard addition. *Mycobacterial tuberculosis* FAs determined were: C19:0, C18:0, C18:1c, C16:0 with C14:0 (also used as an internal standard).

### 2.4. CE with Light-Emitting Diode Detection

Surprisingly, Breadmore *et al.* [58] described the separation of C8-C20 in sesame and sunflower oils derivatised with Nile Blue (NB) as a fluorescent reagent, which produced a high molar absorptivity product by MEKC with SDS micelles and NACE using a red light-emitting diode (LED) [59], as an alternative light source; this is advantageous, since it presents a greater light intensity and a lower noise compared to conventional lamps that are commonly employed in CE. The conditions used were (i) MEKC: 32.5 cm (17.5 cm to the detector) polyimide-coated fused silica capillary, −25 kV, 25 °C, injection at 20 mbar for 3s of 25 µmol L^−1^ of each acid and the BGE consisted of 25 mmol·L^−1^ of phosphate buffer, pH 2.2, 1% w/v of SDS, 7% v/v *n*-butanol, 10% v/v 2-propanol, with separations occurring within about 15 min; (ii) NACE: 72.0 cm (57.5 cm to the detector) polyimide-coated fused silica capillary, +20 kV, 25 °C, sample introduction at 50 mbar for 5 s of 25 µmol L^−1^ of each FA. The BGE consisted of 100 mmol·L^−1^ ammonium acetate, pH 2.2 and 10% v/v glacial acetic acid in acetonitrile, but the separations occurred within about 30 min. However, the efficiency of NACE was superior to MEKC. In addition, the separation of C18:2 and C18:3 was possible using NACE, but was not possible by MEKC.

**Table 2:** Experimental condition, BGE and FA in different samples using UV-ID with different CE modes. NACE: nonaqueous capillary electrophoresis.

Samples	FA	BGE and Experimental Condition	CE Mode and Detection	Year	Ref.
Butter and palm oil	Saturated C_12_ at C_18_	30% acetone, a cationic surfactant and 3,5-dinitrobenzoic acid	CZE-UV-ID	1994	[48]
Butter	Saturated C_8_ at C_20_	10 mmol·L^−1^ SDBS, 50% ACN and 30 mmol·L^−1^ Brij; 23 °C, 20 kV	NACE-UV-ID (λ = 198 nm)	1995	[41]
Coconut oil	Saturated C_2_ at C_18_	20 mmol·L^−1^ Tris, 10 mmol·L^−1^ p-anisate, 1.0 mmol·L^−1^ trimethyl-β-CD in water:methanol 40:60 and pH ~ 8.2; 30 °C, 30 kV	CZE-UV-ID (λ = 270 nm)	1995	[40]
Hydrogenated fish oil	Saturated C_14_ at C_26_	2.5 mmol·L^−1^ antraquinone-2-carboxylic acid and 40 mmol·L^−1^ Tris in NMF-dioxane (3:1, v:v); the temperature stabilized at 6–7 °C above the room temperature, 20 kV	NACE-UV-ID (λ = 264 nm)	1997	[52]
Mix standard	Saturated C_14_ at C_20_	10 mmol·L^−1^ tris, 5 mmol·L^−1^ p- methoxybenzoate at pH 8.1, 10 mmol·L^−1^ Brij in MeOH-H_2_O (50:50, v/v)	MEKC-UV-ID (λ = 254 nm)	1997	[59]
Mix standard	Saturated C_12_ at C_31_	NMF-dioxane (3:2), 40 mmol·L^−1^ Tris, 2.5 mmol·L^−1^ AMP and 0.5% (w/v) Brij; 40 °C, 20 kV	CZE-UV-ID	1999	[46]
Soy and olive oils and fatty acid enriched margarine	C_10_ at C_18:0_, C_16:1c_, C_18:1c_, C_18:2cc_ and C_20_	5.0 mmol·L^−1^ phosphate buffer, 4.0 mmol·L^−1^ SDBS, 4.0 mmol·L^−1^ DM-β-CD and 45% CAN; 25 °C, 25 kV	CZE-UV-ID (λ = 224 nm)	2001	[31]
Parenteral feed formulation, fish oil	C_16:0_, C_18:1c_, C_18:2cc_, C_18:3ccc_, C_20:5ccccc_ and C_22:6cccccc_	5.0 mmol·L^−1^ phosphate buffer, 4.0 mmol·L^−1^ SDBS, 4.0 mmol·L^−1^ DM-β-CD and 45% ACN; 25 °C, 25 kV for ID	CZE-UV-ID (λ = 224 nm for ID)	2003	[36]
Olive, rice and linseed oils and salmon fish	C_16:0_, C_18:0_, C_18:1c_, C_18:2_ and C_18:3_ in the vegetable oils and C_22:5_, C_18:0_, C_18:1_, C_22:6_, C_20:5_ + C_16:0_, C_16:1_ e C_14:0_ in the salmon fish	40 mmol·L^−1^ Tris buffer and 2.5 mmol·L^−1^ AMP in NMF-dioxane-water (5:4:1 v:v:v for vegetable oil FA and 4:6:1 v:v:v for fish oil FA); 25 °C, 25 kV	CZE-UV-ID (λ = 254 nm)	2003	[47]
Hydrogenated of Brazilnut	C_12_ at C_18_ and C_18:1c_, C_18:1t_, C_18:2cc_, C_18:2tt_ and C_18:3ccc_	15 mmol·L^−1^ phosphate buffer, 4.0 mmol·L^−1^ sodium dodecylbenzensulfonate (SDBS), 10 mmol·L^−1^ Brij, 2% 1-octanol, 45% ACN and pH ~ 7.0; 25 °C, 20 kV	CZE-UV-ID (λ = 224 nm)	2003	[53]
Olive oil	C_18: 1c ,_C_18:2cc _and C_16:0_	15 mmol·L^−1^ phosphate buffer solution at pH 6.86 containing 4 mmol·L^−1^ SDBS, 10 mmol·L^−1^ Brij 35, 45% v/v ACN and 2% v/v 1-octanol; 25 °C, 20 kV	CZE-UV-ID (λ = 224 nm)	2007	[43]
Peanut seeds	C_18.1_ and C_18.3_	40 mmol·L^−1^ Tris, 2.5 mmol·L^−1^ AMP and 7.0 mmol·L^−1^ α-CD in NMF-dioxane-water (5:3:2), pH 8–9; 25 °C, 28 kV	CZE-UV-ID (λ = 254 nm)	2008	[27]
Hydrogenated vegetable fat and spreadable cheese	C_18:1c_ and C_18:1t_	15.0 mmol·L-1 KH_2_PO_4_/Na_2_HPO_4_ buffer (pH∼7.0), 4.0 mmol·L-1 SDBS, 8.0 mmol·L-1 Brij35, 45%v/v ACN, 8% methanol, and 1.5% v/v n-octanol; 25 °C, 28 kV	CZE-UV-ID (λ = 224 nm)	2010	[42]
Oils from avocado, corn, extra virgin olive, hazelnut, and soybean	C _18:0;,_ C _18:1_; C _16:0_; , C_18:2_; C _16:1_; C _18:3_ and C _14:0_	10 mmol·L^−1^ p-hydroxybenzoate, 5 mmol·L^−1^ Tris, 80 mmol·L^−1^ Brij 98, 40% acetonitrile, 10% 2-propanol and pH 8.8; 45 °C, 25 kV	CZE-UV-ID (λ = 254 nm)	2011	[51]
Cosmetic formulations containing Brazilian nut oil	C_18.0,_ C_17.0,_ C_16.0,_ C_18.1c_, C_18.2cc_ and C_18.3_	12.5 mmol·L^−1^ sodium tetraborate buffer, 12.5 mmol·L^−1^Brij, 7.5 mmol·L^−1^SDBS and 35% ACN pH 7.0; 25 °C, 20 kV	CZE-UV-ID (λ = 254 nm)	2011	[45]
Spreadable cheese	C_18: 2 cis -9 cis-12, _C_18: 1 cis- 9,_ C_18: 1 trans-9, _C_18.0_	15 mmol·L^−1^ NaH_2_PO_4_/Na_2_HPO_4_ at pH 6.86, 4 mmol·L^−1^ SDBS , 8.3 mmol·L^−1^ Brij 35, 45% v/v ACN and 2.1% n-octanol; 25 °C, 19 kV	CZE-UV-ID (λ = 224 nm)	2012	[28]
*Brachiaria ruzizienses* forages	C_18.2 n-6 _and C_18.3 n-3_	15 mmol·L^−1^ NaH_2_PO_4_/Na_2_HPO_4_, 4.0 mmol·L^−1^ SDBS , 10 mmol·L^−1^ Brij 35, 43.5% ACN and 2.2% of 1-octanol; 25 °C, 19 kV	CZE-UV-ID (λ = 224 nm)	2013	[30]
Soybean oil, olive oil, butter, margarine, cookie filling, hydrogenated vegetable fat and beef liver	C_18,0_,C_18.1t,_ C_18.1c,_C_16.0_, C_18.2cc_ , C_13.0_ and C_18.3ccc_	15 mmol·L^−1^ NaH_2_PO_4_/Na_2_HPO_4_ at pH 6.86, 4.0 mmol·L^−1^ SDBS , 8.3 mmol·L^−1^ Brij 35, 45% v/v ACN and 2.1% of 1 – octanol; 25 °C, 19 kV	CZE-UV-ID (λ = 224 nm)	2013	[44]
Soybean oil, virgin and extra virgin olive oil	C_18: 2cc,_ C_18:1c,_ C_16:0_	15 mmol·L^−1^ NaH_2_PO_4_/Na_2_HPO_4_, 4.0 mmol·L^−1^ SDBS , 10 mmol·L^−1^ Brij 35, 43.5% ACN and 2.2% of 1-octanol; 25 °C, 19 kV	CZE-UV-ID (λ = 224 nm)	2014	[54]
Soybean oil and rat liver	C_18:2cc,_ C_18:1c,_C_18:0,_ C_16.0,_ C_18:3ccc_	15 mmol·L^−1^ NaH_2_PO_4_/Na_2_HPO_4_, 4.0 mmol·L^−1^ SDBS , 10 mmol·L^−1^ Brij 35, 43.5% ACN and 2.2% of 1-octanol; 25 °C, 19 kV	CZE-UV-ID (λ = 224 nm)	2014	[55]

### 2.5. CE with Capacitively Coupled Contactless Conductivity Detection

Conductivity detection has received considerable attention as an alternative detection method in CE. In capacitively coupled contactless conductivity detection (C^4^D) measurements, an AC-voltage is applied to one of the galvanically isolated electrodes and the resulting AC-current is measured at the second electrode. This is possible as the electrodes form capacitors with the electrolyte solution, which are transparent for AC-signals [60]. Besides, under normal conditions, the C^4^D offers superior sensitivity in comparison with indirect detection by UV absorption, with the extra advantage of not requiring the opening of detection windows in the capillary, increasing its durability and facilitating its use [61].

The first article found to address the analysis of FAs using CE-C^4^D was by Oliveira *et al.* in 2003 [62]*.* The electrolyte used was composed of 5.0 mmol·L^−1^ of phosphate buffer at pH 7, 4.0 mmol·L^−1^ of dimethyl-β-cyclodextrin, 2.0 mmol·L^−1^ of trimethyl-β-cyclodextrin, 50% (v/v) of ACN, and 20% (v/v) of methanol. Analysis conditions were normal polarity, voltage +20 kV, sample introduction by gravity at 10 cm for 10 s and bare fused-silica capillary (75 μm internal diameter), with total and effective lengths of 60 cm and 50 cm, respectively. Under these conditions, using a mixture of standards, the following FAs were determined: C8:0, C9:0, C10:0, C11:0, C12:0, C14:0, C16:0, C18:0, C20:0. In a sample of coconut vegetable oil, the following FAs were identified: C8:0, C10:0, C12:0, C14:0, C18:2, C18:1, C16:0, C18:0. In this case, the unsaturated FAs were determined by the fortification of the sample; C18: 1 and C16:0 migrated together, and were identified in the same peak. The total analysis time was 16 min. The sample preparation for coconut vegetable oil was done by saponification followed by its dilution in methanol, and solutions of each FA were prepared in methanol.

LCFA were determined in recent and aged samples of drying oils, used as binding media for objects of art, and in samples taken from two paintings of the 19th century by Surowiec *et al.* [63] in 2004. These authors simultaneously used two detections, contactless conductivity and UV-ID detection, and also determined dicarboxylic acids. However, only C^4^D results described for LCFAs will be shown. The electrolyte was optimised as 15 mmol·L^−1^ of phosphate buffer at pH 6*.*86, 4 mmol·L^−1^ of SDBS, 10 mmol·L^−1^ of Brij 35, 1-octanol 2% (v/v), and ACN 45% (v/v). Analysis conditions were normal polarity, voltage +20 kV, temperature at 25 °C, hydrodynamic sample introduction of 75 mbar and fused-silica capillary (75 μm internal diameter), total and effective lengths of 48.5 cm and 33.4 cm, respectively. The FAs determined with standards were: C18:0, C18:1, C16:0, C18:2, C18:3. The same FAs were found in samples of recent and aged linseed oil. A walnut oil sample did not show the peak of C18:3 to be well resolved, despite having been possibly identified. A sample taken from a painting from the 19th century presented C18:0, C16:0 and C18:3. Analysis times ranged from 7 and 8.5 min. The sample preparation was performed by common hydrolysis followed by extraction from acidic solution with diethyl ether. 

Recently, Buglione *et al.* [64] determined SCFAs and LCFAs using NACE-C^4^D. Another difference was the use of a fused silica capillary coated with poly(vinyl alcohol) (PVA). The optimised electrolyte composition was buffer, 10 mmol·L^−1^ of deoxycholate and 100% of methanol. Analysis conditions for standards were reversed polarity, voltage −25 kV, hydrodynamic injection for 0.2 min at 50 mbar and PVA-coated capillary (75 μm internal diameter), and total and effective lengths of 40 and 25 cm. The FA determined with standards were: C3:0, C4:0, C5:0, C6:0, C7:0, C8:0, C9:0, C10:0, C11:0, C12:0, C14:0, and C16:0. Analysis conditions for edible oils were reversed polarity, voltage −30 kV, hydrodynamic sample introduction for 0.1 min at 50 mbar and PVA-coated capillary (75 μm internal diameter), with total and effective lengths of 55 and 40 cm. The FAs determined in homemade olive, commercial olive oil and sunflower oil were: C16:0, C18:2, C18:1, and C18:0. The presence of the last three FAs explains the need for a slightly longer capillary. The identification of these FAs was performed by matching migration times with further standards. Total analysis time was approximately 11 min. Sample preparation was performed by dissolving the samples in 1:1 methanol/dichloromethane mixture and solutions of each FA were prepared in methanol.

Another recent study that applied CZE-C^4^D was the one by Wong *et al.* [3]. This paper presents an optimization of BGE in which variables such as pH, use of cyclodextrins, and effect of organic modifiers, among others were investigated. The optimised electrolyte composition was 6.0 mmol·L^−1^ of methyl-β-cyclodextrin, 8.0 mmol·L^−1^ of heptakis-(2,3,6-tri-O-methyl)-β-cyclodextrin, 5.0 mmol·L^−1^ of Na_2_HPO_4_/KH_2_PO_4_ at pH 7.4, 40% (v/v) of ACN, 25% (v/v) of methanol and 5% (v/v) of 1-octanol. Analysis conditions were normal polarity, voltage +30 kV, temperature at 20 °C, hydrodynamic injection at 50 mbar s^−1^ and bare fused-silica capillary (75 μm internal diameter), with total and effective lengths of 80.5 cm and 70 cm. From the optimised conditions, it was possible to separate these FAs into standards: C20:0, C18:0, C18:1t, C18:1c, C16:0, C18:2, C15:0, C18:3, C14:0, C12:0. The applicability of the method was assessed using samples of butter, and the following FAs were determined: C18:0, C18:1t, C18:1c, C16:0, C18:2, C18:3, C14:0, C12:0. The total analysis time was 36 min. Butter samples were prepared by saponification followed by dilution in MeOH.

### 2.6. CE with Mass Spectrometric Detection

CE-MS combines the advantages of CE and mass spectrometry (MS); therefore, high separation efficiency and molecular masses or fragmentation can be obtained on the analyses by differences in electrophoretic mobility and structural information [65]. Several ionisation methods have been used in CE-MS, such as electrospray ionisation, ion spray and continuous-flow fast atom bombardment. The combination of CE-MS and electrospray ionisation (ESI) is a concentration-sensitive detection method, the sensitivity of which will suffer from dilution by sheath-flow.

Petersson *et al.* [66] described a new sheathless electrospray interface between CE and MS, with ESI in the negative mode to analyse four FAs (lauric acid, myristic acid, palmitic acid and stearic acid), and six prostaglandins. The FAs were dissolved in the electrolyte which consisted of 2 mmol·L^−1^ acetic acid ammonium acetate in 50% of ACN in water at pH 5.2, with sample introduction at 2 kPa for 5 s and the applied voltage over a 50 cm fused-silica capillary of +32 kV. The detection was performed by selected ion recording of the FA. The FA separation occurred within 3 min in a laboratory-built CE system and a Micromass Quattro-LC equipped with a Z-spray ion source as a detector.

Summary information about CE-LIF, CE-C^4^D and CE-MS for FA analysis described in Section 2.3 to Section 2.6 is presented in Table 3.

**Table 3:** Experimental condition, BGE and FA in different samples using LIF, LED, C^4^D and MS detection with different CE modes.

Samples	FA	BGE and Experimental Condition	CE Mode and Detection	Year	Ref.
Mix standard	C_3:0_, C_5:0_, C_6:0_, C_10:0_, C_12:0_, C_18:1_ and C_16:0_	methanol with 12.5 mmol·L^−1^; tetraethylammonium chloride; ambient temperature, 25 kV	CE-LIF (λ = 780 nm)	1999	[56]
Mycobacterium tuberculosis fatty acids	C_19:0_, C_18:0_, C_18:1c_, C_16:0_ with C_14:0_ (internal standard)	25 mmol·L^−1^ aqueous sodium borate and acetonitrile 30% (v/v) at pH 9; 25 °C, 30 kV	CE-LIF (λ = 488 nm)	2002	[57]
Mix standard	Saturated C_8_-C_20_	25 mmol·L^−1^ phosphate buffer, pH 2.2, 1% w/v SDS, 7% v/v *n*-BuOH, 10% v/v 2-propanol; 25 °C, −25 kV for MECK and 100 mM ammonium acetate, pH 2.2, 10% v/v glacial acetic acid in ACN; 25 °C, +20 kV for NACE	MECK-LED and NACE-LED	2007	[58]
Coconut vegetable oil	C_8:0_, C_10:0_, C_12:0_, C_14:0_, C_18:2_, C_18:1_, C_16:0_ and C_18:0_	5.0 mmol·L^−1^ phosphate buffer at pH 7, 4.0 mmol·L^−1^ dimethyl-β-cyclodextrin, 2.0 mmol·L^−1^ trimethyl-β-cyclodextrin, acetonitrile 50% , and methanol 20%; 20 kV	CE-C^4^D	2003	[62]
Recent and aged linseed oil, aged walnut oil and oil paintings from the 19th century	C_18:0_, C_18:1_, C_16:0_, C_18:2_ and C_18:3_	15 mmol·L^−1^ phosphate buffer at pH 6.86, 4 mmol·L^−1^ SDBS, 10 mmol·L^−1^ Brij 35, 1-octanol 2% and acetonitrile45%; 25 °C, 20 kV	CE-C^4^D	2004	[63]
Homemade olive oil, commercial olive oil and sunflower oil	C_16:0_, C_18:2_, C_18:1_ and C_18:0_	buffer, 10 mmol·L^−1^ deoxycholate and 100% methanol; −30 kV	NACE-C^4^D	2013	[64]
Margarine	C_18:0_, C_18:1t_, C_18:1c_, C_16:0_, C_18:2_, C_18:3_, C_14:0_ and C_12:0._	6 mmol·L^−1^ methyl-β-cyclodextrin, 8 mmol·L^−1^ heptakis-(2,3,6-tri-O-methyl)-β-cyclodextrin, 5 mmol·L^−1^ Na_2_HPO_4_/KH_2_PO_4_ at pH 7.4, acetonitrile 40%, methanol 25% and 1-octanol 5%; 20 °C, 30 kV	CE-C^4^D	2013	[3]
Mix standard	Saturated C_12_, C_14_, C_16_ and C_18_	2 mmol·L^−1^ acetic acid ammonium acetate in 50% (v/v) acetonitrile in water at pH 5.2; 32 kV	CZE-MS	1999	[66]

## 3. Conclusions

In the present review, the situation since 1994 regarding FA analysis by CE using different modes such as CZE, MECK, NACE, and MEEKC, associated with different detection systems such as UV, LED, LIF, C^4^D and MS, was described. Several BGE approaches, taking into account buffers fitted with pH equal to or higher than 6.5, with the addition of organic solvent, cyclodextrin and surfactant, were discussed. In general, CE approaches present low cost, short analysis time, higher throughput, simple sample preparation steps and the absence of the use of specific columns for the separation of *cis-trans* homologues as an advantage in comparison with the classical gas chromatography (GC) methodology. On the other hand, CE presents low sensibility and a limited range of FA analysis in comparison with GC. However, through the present review, it could be demonstrated that CE seems to be an interesting analytical separation technique that is very useful for screening analysis or quantification of the usual FAs present in different matrixes, which may be useful to help with quality control in industry and government agencies, as both the sample preparation and analysis time are very attractive. Finally, the authors believe that the present review could be a very useful reference source for researchers, students, and users interested in the use of CE for FA analysis, since key information has been described adequately.

## Supplementary Materials

Supplementary materials can be accessed at: http://www.mdpi.com/1420-3049/19/9/14094/s1.

## Supplementary Files

Supplementary File 1
