# Supplementary material for: 20 Years of Fatty Acid Analysis by Capillary Electrophoresis
Source: Molecules. 2014 Sep 9;19(9):14094–113. doi: 10.3390/molecules190914094 (PMC6270880; doi:10.3390/molecules190914094)
Supplement: Supplementary File 1 [file molecules-19-14094-s001.pdf]

Supplementary Material

Capillary Electrophoresis: Conceptual, Fundamentals and Instrumentation

Electrophoresis was introduced by Tiselius in the beginning of the 1930s, through demonstration of the elegant method called mobile border, which demonstrated the partial separation of some protein constituents of blood serum. As a result of this pioneering work, the Nobel Award of 1948 was granted to Tiselius. Since then, electrophoresis has occupied a featured place among the methodologies used in biomolecules analyses. However, from the middle of the 1980s, through the implementation of capillaries techniques, electrophoresis has been developed from intensive manipulation to an automated format, deserving the status of a routine analytical technique [1,2].

Conceptually, CE is a separation technique that operates in liquid medium (aqueous or organic), which is based on the differentiated migration of neutral, ionic or ionisable compounds, when an electrical field in the order of kilovolts is applied tangentially to capillary tubes containing a background electrolyte (BGE) such as a buffer or a simple electrolyte solution [3,4]. When the electric field is applied, the compounds migrate with constant velocity and are separated into distinct zones, crossing the detector system that is conveniently positioned in a defined section of the capillary column. In general, the analyte separations are related to simultaneous contributions between electrophoretic mobility and electroosmotic flow (to be considered in the next paragraph). The electrophoretic mobility is an intrinsic characteristic of analytes and is dependent upon the charge of the molecule, the viscosity and the effective radius of the species. Overall, the electrophoretic mobility will be dependent of the size/charge relationship. Thus, if two ions are the same size, the one with the highest charge will move fastest because of the higher charge/size relationship. In the same way, for ions containing the same charge, the smallest one has less friction and a faster migration rate because this presents a higher charge/size relation [5,6]. On the other hand, neutral species will migrate according to EOF velocity. Consequently, different electromigration techniques in capillaries containing singular mechanisms and characteristic selectivity are possible by CE, such as [7]:

- Capillary zone electrophoresis (CZE) or free solution capillary electrophoresis (FSCE), which is a separation technique based on differences between the mobility of charged species (analytes), in an aqueous or organic BGE. The BGE can contain additives such as cyclodextrins, complexants or ligands which are able to interact with analytes and change their electrophoretic mobility;
- Capillary affinity electrophoresis (CAE) or affinity capillary electrophoresis (ACE) is an electrophoretic separation technique in which the compounds are able to specifically interact with analytes added to the BGE;
- Capillary sieving electrophoresis (CSE) is an electrophoretic separation technique involving a sifter (for example: tangled network of linear polymers) contained within BGE. The separation is based on differences in size and shape between the charged analytes;
- Capillary gel electrophoresis (CGE), a special case of sieving capillary electrophoresis in which the capillary is filled with gel containing *cross-linked*;
- Capillary isoelectric focusing (CIEF), an electrophoretic technique for separating amphoteric analytes according to their isoelectric point, by applying an electric field along a pH gradient generated in the capillary;

- Capillary isotachophoresis (CITP) is an electrophoretic separation technique performed on a system with a discontinuous electrolyte, wherein the analytes migrate according their electrophoretic mobility, forming a chain of adjacent areas that move with equal velocity between two solutions, the electrolyte leader and terminator. The conditions that must be met for the analytes to migrate in adjacent zones are the mobility of the ions of the leading electrolyte and terminator being higher and lower, respectively, than the mobility of the ions in the sample [8];
- Electrokinetic chromatography (EKC) or electrokinetic capillary chromatography (ECC), a separation technique based on the combination of electrophoresis and interactions of the analytes with additives (for example: surfactant) to form a dispersed phase that moves with different speeds of the analyte. In order to promote separation, the analytes or dispersed phase should possess a charge;
- Micellar electrokinetic chromatography (MEKC) or micellar electrokinetic capillary chromatography (MECC), a special case of electrokinetic chromatography wherein the phase dispersed in the capillary is the micelle [9];
- Microemulsion electrokinetic chromatography (MEEKC) or microemulsion electrokinetic capillary chromatography (MEECC), a special case of electrokinetic chromatography, in which a microemulsion is used as the dispersed phase;
- Capillary electrochromatography (CEC), a special case of capillary liquid chromatography, in which the drive of the mobile phase through a capillary filled or coated with the stationary phase is promoted by the electroosmotic flow (which may be assisted by pressure). Retention time is determined by a combination of electrophoretic migration and the chromatographic retention [10];
- Non-aqueous capillary electrophoresis (NACE), a separation technique based on differences among the analyte mobility using an organic BGE. Non-aqueous media have been considered particularly suitable for compounds that are not readily soluble in water and compounds that show very similar electrophoretic mobility in aqueous media [11].

In general, the capillaries consist of fused silica (relevant resistance to chemical attack) and present very well-defined dimensions, that is, 100, 75 and 50 micrometres are the most typical inner diameters and 360 μm is the outside diameter (high internal surface area relative to volume favours the dissipation of heat generated by the Joule effect), containing a polymeric external coating (polyimide, polyacrylate, fluoropolymer), which bestows the tube with mechanical strength and flexibility. However, it is also possible to use capillary tubes made of other materials such as glass, Teflon and Peek. Due to the intrinsic propriety of the inner wall of the fused silica capillaries, that is, the presence of silanol groups, an important phenomenon called electroosmotic flow (EOF) is present. At a pH higher than 3.0, ionisation of the silanols groups from the inner capillary wall occurs, resulting in a negative charge being acquired and a counter-ion layer being formed near the capillary wall, generating a neutral double electric layer. When high positive voltage is applied in the inlet, electric powers acting in the capillary wall surface and the ions drag water molecules, creating an EOF which is directed to the cathode, characterising a normal EOF inside the capillary. On the other hand, anodic EOF is possible when a quaternary ammonium salt is added to the buffer solution, in order to generate

a dynamic recoating in the inner capillary wall with a positive charge, associated with the application of high negative voltage in the inlet. This results in a reverse EOF inside the capillary, which is very useful for fast anion analysis, that is, those containing high charge and smaller diameters. In general, the EOF can be influenced by the pH, viscosity and BGE ionic strength, as well as the applied voltage [3,12]. Another interesting feature of a fused silica capillary is the wide optical window, making its use attractive and versatile for different applications. In summary, in CE, it is possible to work with several detection approaches, that is, the same used for high performance chromatography (HPLC). Thus, among the detection systems used to CE, the following can be highlighted as more usual: ultraviolet-visible detection (UV-Vis), capacitively coupled contactless conductivity detection (C^4D), Laser-induced fluorescence detection (LIF), and mass spectrometry (MS). Finally, another interesting feature associated with the tube capillary is the reduced volume of BGE or sample (of the order of nanolitres) that is necessary to perform the analysis.

The CE equipment, in general, consists of vials of solutions (standard, sodium hydroxide, water, BGE, samples, *etc.*), a carousel for the vials, a high voltage power supply, a cartridge under controlled temperature, where the capillary tube is fixed inside (it helps in the dissipation of heat generated by Joule effect), a pair of platinum electrodes and a detection system (for example, diode array detection), as shown in the simplified scheme in Figure S1. All devices are connected to a microcomputer containing software that is able to perform control, acquisition and treatment of data. Depending on the sample features or the CE mode used, the samples can be introduced into the capillary by the application of pressure (hydrodynamic) or voltage (electrokinetic) within a defined time interval.

Figure S1. Simplified scheme of a CE instrument.

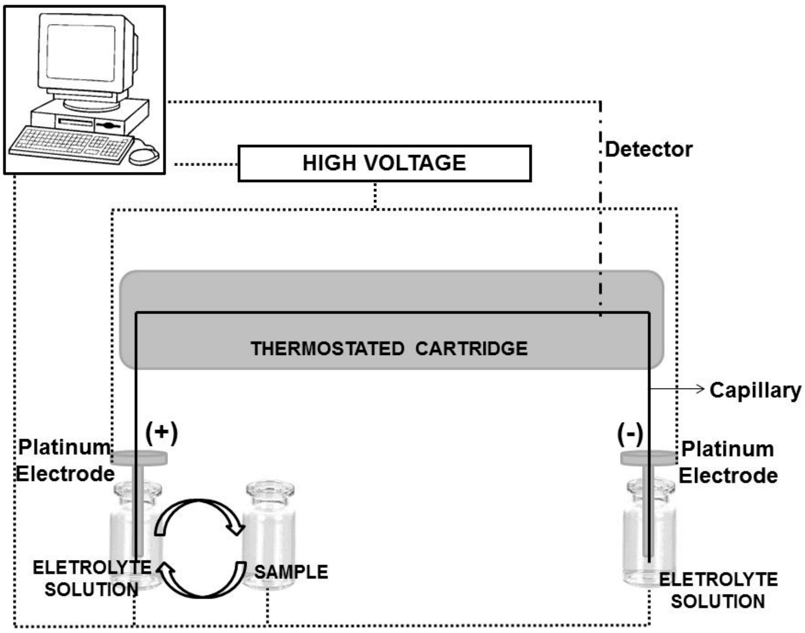

References

1. Tiselius, A. *The Moving Boundary Method of Studying the Electrophoresis of Proteins*; University of Uppsala: Uppsala, Sweden, 1930.

2. Tiselius, A. A new apparatus for electrophoretic analysis of colloidal mixtures. *Trans. Faraday Soc.* **1937**, *33*, 524–531.
3. Tavares, M.F.M. Eletroforese capilar: Conceitos básicos. *Quim. Nova* **1996**, *19*, 173–181.
4. Dabek-Zlotorzynska, E.; Lai, E.P.C.; Timerbaev, A.R. Capillary electrophoresis: The state of the art in metal speciation studies. *Anal. Chim. Acta* **1998**, *359*, 1–26.
5. Baker, D.R. *Capillary Electrophoresis*; Wiley: Hoboken, NJ, USA, 1995.
6. Li, S. *Capillary Electrophoresis: Principles, Practice and Applications*; Elsevier Science Publishers: Amsterdam, The Netherlands, 1992.
7. Riekkola, M.L.; Jönsson, J.Å.; Smith, R.M. Terminology for analytical capillary electromigration techniques (IUPAC Recommendations 2003). *Pure Appl. Chem.* **2004**, *76*, 443–451.
8. Weston, A.; Brown, P. *HPLC and CE: Principles and Practice*; Academic Press: San Diego, CA, USA, 1997.
9. Terabe, S.; Matsubara, N.; Ishihama, Y.; Okada, Y. Microemulsion electrokinetic chromatography—Comparison with micellar electrokinetic chromatography. *J. Chromatogr. A* **1992**, *608*, 23–29.
10. Ceccon, L.; Saccu, D.; Procida, G.; Cardinali, S. Liquid chromatographic determination of simple phenolic compounds in waste waters from olive oil production plants. *J. Aoac Int.* **2001**, *84*, 1739–1744.
11. Sahota, R.S.; Khaledi, M.G. Nonaqueous Capillary Electrophoresis. *Anal. Chem.* **1994**, *66*, 1141–1146.
12. Kuhn, R.; Hoffstetter-Kuhn, S. *Capillary Electrophoresis: Principles and Practice*; Springer-Verlag: Berlin, Germany, 1993.
